# Introducing a new type of alternative laryngeal mucosa model

**DOI:** 10.1371/journal.pone.0287634

**Published:** 2023-06-30

**Authors:** Tanja Grossmann, Andrijana Kirsch, Magdalena Grill, Barbara Steffan, Michael Karbiener, Luka Brcic, Barbara Darnhofer, Ruth Birner-Gruenberger, Markus Gugatschka

**Affiliations:** 1 Department of Otorhinolaryngology, Division of Phoniatrics, Medical University of Graz, Graz, Austria; 2 Diagnostic and Research Institute of Pathology, Diagnostic and Research Center of Molecular Medicine, Medical University of Graz, Graz, Austria; 3 BioTechMed-Graz, The Omics Center Graz, Graz, Austria; 4 Institute of Chemical Technologies and Analytics, Technische Universität Wien, Vienna, Austria; University of Cincinnati, UNITED STATES

## Abstract

Research of human vocal fold (VF) biology is hampered by several factors. The sensitive microstructure of the VF mucosa is one of them and limits the *in vivo* research, as biopsies carry a very high risk of scarring. A laryngeal organotypic model consisting of VF epithelial cells and VF fibroblasts (VFF) may overcome some of these limitations. In contrast to human VFF, which are available in several forms, availability of VF epithelial cells is scarce. Buccal mucosa might be a good alternative source for epithelial cells, as it is easily accessible, and biopsies heal without scarring. For this project, we thus generated alternative constructs consisting of immortalized human VF fibroblasts and primary human buccal epithelial cells. The constructs (n = 3) were compared to native laryngeal mucosa at the histological and proteomic level. The engineered constructs reassembled into a mucosa-like structure after a cultivation period of 35 days. Immunohistochemical staining confirmed a multi-layered stratified epithelium, a collagen type IV positive barrier-like structure resembling the basement membrane, and an underlying layer containing VFF. Proteomic analysis resulted in a total number of 1961 identified and quantified proteins. Of these, 83.8% were detected in both native VF and constructs, with only 53 proteins having significantly different abundance. 15.3% of detected proteins were identified in native VF mucosa only, most likely due to endothelial, immune and muscle cells within the VF samples, while 0.9% were found only in the constructs. Based on easily available cell sources, we demonstrate that our laryngeal mucosa model shares many characteristics with native VF mucosa. It provides an alternative and reproducible *in vitro* model and offers many research opportunities ranging from the study of VF biology to the testing of interventions (e.g. drug testing).

## Introduction

The laryngeal mucosa is a highly specified tissue with characteristics found nowhere else in the human body. Located at the intersection of the respiratory and digestive tract, it serves as the primary sound generator, subjecting it to considerable mechanical forces. At the same time, it is exposed to a number of noxae, of which cigarette smoke and air pollution are the most prominent. Together, these factors make the laryngeal mucosa a vulnerable structure that can lead to benign and malignant disease.

The small size of the vibrating part of the human vocal fold (VF) mucosa and its delicate microarchitecture severely limit its *in vivo* study, as biopsies of healthy VF mucosa are virtually impossible. Furthermore, our understanding of VF biology, laryngeal diseases (benign and malignant), and consequently of treatment options other than surgery, is also insufficient.

Recent developments include alternatives to widely used animal studies, namely the construction of organotypic laryngeal mucosa models, consisting of laryngeal epithelial cells and fibroblasts [1,2]. Ideally, these 3D cell constructs will serve as models to study both physiological (e.g. the interaction of the epithelium and the lamina propria) and pathophysiological questions (e.g. the impact of cigarette smoke or allergens on the mucosa). Ultimately, these organotypic models may pave the way for the bioengineering of autologous grafts for tissue replacement.

While human vocal fold fibroblasts (hVFF) are principally available (e.g. the mucosa can be harvested during arytenoidectomy or from postmortem biopsies), human VF epithelium which is required for these experiments, is only available in exceedingly small numbers [3], so alternatives for this cell type are urgently needed. Epithelial cells of buccal mucosal origin could be such a candidate with similar properties, as they are easy to harvest, available in sufficient numbers, and biopsies heal without scarring [4].

The aim of our study was thus to engineer constructs consisting of human buccal epithelial cells (hBEC) and immortalized hVFF *in vitro* and to compare these constructs to native laryngeal mucosa at the histological and proteomic level. Given a high similarity to laryngeal mucosa, this would enlarge the repertoire in laryngeal research significantly for the above mentioned questions. The abundant availability of both cell types makes the 3D model an interesting and alternative *in vitro* research tool for other groups working in this field.

## Materials and methods

### Cell types, tissues and constructs

#### Human buccal epithelial cells (hBEC)

Human buccal mucosa (BM) samples (n = 4, mean age ± SD = 50 ± 4.3, 2 male, 2 females, for donor demographic see Table 1) were collected during routine surgeries after written informed consent of patients and approval of the local ethics committee (approval number 27–396 ex 14/15). In detail, tissue pieces (Ø ~6 mm) from unsuspicious mucosa were collected in decontamination medium (DM) consisting of high glucose Dulbecco’s modified Eagle’s medium (DMEM, Sigma Aldrich, Vienna, AT), supplemented with 1% Antibiotic-Antimycotic solution (Thermo Fisher Scientific, Waltham, Massachusetts, USA) and 100 μg/ml Normocin (Invivogen, San Diego, CA, USA), and immediately decontaminated for 10 minutes in fresh DM. Then, pieces were digested in a Dispase-solution (1.2 U/ml; Thermo Fisher Scientific) at 4°C for 15 hours. After incubation, the epithelial layer was separated carefully from the underlying stroma and digested in an Accutase-solution (500–720 U/ml, Sigma Aldrich) for 10–15 minutes at 37°C. Thereafter, the solution was carefully pipetted up and down and the resulting single-cell solution was transferred into a 15 ml Falcon and diluted with 6 ml of CnT-PR medium (CellnTec Advanced Cell Systems AG, Bern, Switzerland). The sample was centrifuged at 180 x g for 5 minutes at room temperature, the supernatant was discarded and the cell pellet was diluted in fresh CnT-PR medium supplemented with IsoBoost (50 μl/50 ml, CellnTec Advanced Cell Systems AG), and transferred into wells of a 12-well plate (1 ml/well). Cell cultivation was performed under standard cell culture conditions (37°C, 5% CO_2_) using CnT-PR medium supplemented with IsoBoost for 5 days with daily medium change, followed by a change to CnT-PR medium without supplement with partial medium change every 2–3 days. Adherent growing hBEC were passaged depending on the density of confluence in a ratio of 1:2 using Accutase-solution (50 μl/cm^2^). Cells from all four isolated hBEC (hBEC 1–4) were harvested at passage number 2 for RNA isolation and Reverse Transcription-quantitative Polymerase Chain Reaction (RT-qPCR) to perform a descriptive cell-type specific characterization before they were further cultivated and used for further experiments.

**Table 1:** Donor demographic of isolated hBEC.

Sample	Sex	Age
hBEC 1	female	51
hBEC 2	female	44
hBEC 3	male	49
hBEC 4	male	56

#### Immortalized human vocal fold fibroblasts (hVFF)

Immortalized hVFF [5], kindly provided by Prof. Susan Thibeault, University of Wisconsin-Madison, were cultured under standard cell culture conditions in standard medium (SM) consisting of DMEM supplemented with 10% fetal bovine serum (FBS, Biowest, Nuaillé, FR) and 100 μg/ml Normocin. Adherent growing cells were passaged depending on the density of confluence using 0.5% Trypsin/EDTA (Sigma Aldrich).

#### Human vocal fold (VF) and buccal mucosa (BM) tissue samples

Human VF tissue samples including the underlying vocalis muscle tissue and BM samples (n = 3, mean age ± SD = 60 ± 8.6, for donor demographic see Table 2) were collected from cadavers during autopsy at the Diagnostic and Research Institute of Pathology at the Medical University of Graz. The participant consent was waived by the local ethics committee (approval number 29–036 ex 16/17). Samples were collected within 6 hours after death of patients without any intubation. After decontamination in DM, sections of samples were fixed in 4% paraformaldehyde (PFA, Gatt-Koller, Absam, Austria) for 24 hours, embedded in paraffin using the Tissue Tek VIP (Sakura Finetek USA, Torrance, California, USA) automated embedding machine and cut in 5 μm sections for histological analysis. Further sections of samples were stored at -80°C until further use for proteomic analyses.

**Table 2:** Donor demographic of native VF and BM tissue samples.

Sample	Sex	Age	Cause of death
VF 1/BM 1	female	48	hepatorenal syndrome
VF 2/BM 2	female	68	cardiac arrest
VF 3/BM 3	female	64	cardiac arrest

#### 3D co-cultivation of hBEC and hVFF

To test the appropriate seeding number of fibroblasts, hVFF at different concentrations (5x10^4^, 1x10^5^, and 2x10^5^ cells/insert) were embedded within rat tail collagen, type I matrix (final concentration 2.4 mg/ml, Thermo Fisher Scientific) according to the manufacturer´s instructions in 12-well Transwell inserts (Corning Transwell, polyester membrane, 0.4 μm pore size, Sigma Aldrich) and cultivated for 14 days with partial medium change three times a week. After cultivation, samples were fixed in 4% PFA and allocated to histological H&E staining as described below to visualize hVFF contribution and density.

**Fig 1:**
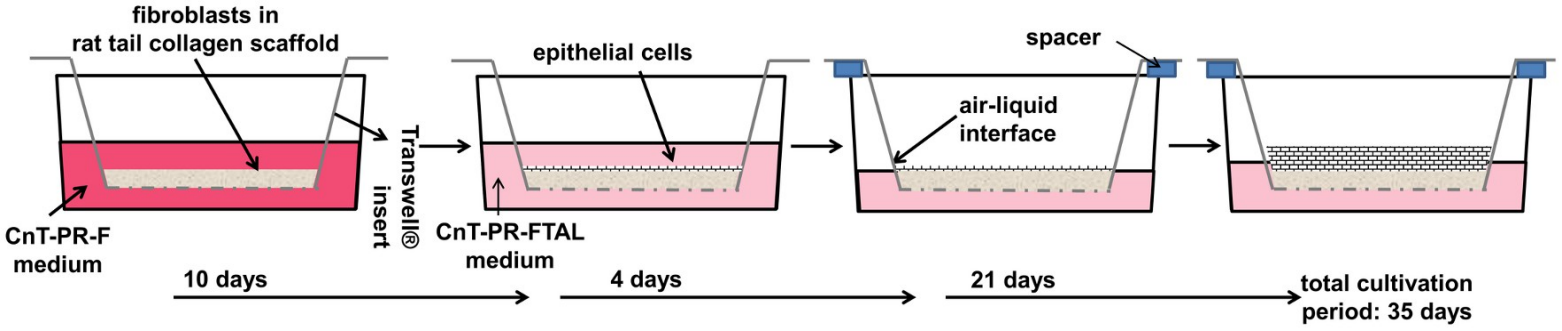
Schematic illustration of the 3D co-cultivation procedure. Fibroblast-specific culture medium (CnT-PR-F), fully defined co-culture medium (CnT-PR-FTAL).

For 3D co-cultivation with isolated epithelial cells, hVFF at a concentration of 2x10^5^ cells in 720 μl SM per insert were embedded within rat tail collagen, type I, as tested previously. Polymerization was performed for 45 minutes at 37°C. Thereafter, fibroblast-specific medium CnT-PR-F (CellnTec Advanced Cell Systems AG) was added to the apical and basolateral sides of the inserts and cultivation was done under standard cell culture conditions for additional 10 days with partial medium change twice a week. Then, cells from hBEC 1 were used to perform the first generation of 3D co-cultivation experiments and seeded at a density of 2x10^5^ cells on top of the established fibroblast-collagen matrix. Histological analysis of technical duplicates for these experiments showed, that cell density was insufficient. Cells from hBEC (hBEC 2–4) were seeded on top of the established fibroblast-collagen matrix at a density of 2.4x10^5^ cells in 600 μl of fully defined co-culture medium CnT-PR-FTAL (CellnTec Advanced Cell Systems AG) per insert in technical duplicates. CnT-PR-FTAL medium was added to both sides of the inserts and constructs were further cultivated for 4 days with medium change on day two. Then, sterilized plate spacers were placed on top of new culture plates and inserts were carefully transferred into new plates. CnT-PR-FTAL medium was fully aspirated from the apical side of the inserts to create the so-called “air-liquid interface” (ALI) and constructs were cultivated for additional 21 days with partial medium change of CnT-PR-FTAL medium in the basolateral sides of the inserts twice a week under standard cell culture conditions (schematic illustration of procedure see Fig 1). Thereafter, co-cultivated constructs (CC) (n = 3, 3 biological replicates in technical duplicates) were harvested for subsequent histological and proteomic analyses.

### Analyses

#### RNA isolation and Reverse Transcription-quantitative Polymerase Chain Reaction (RT-qPCR)

For descriptive cell-type specific characterization, cultivated hBEC from 4 different donors (hBEC 1–4, passage number 2), human keratinocyte cell line (HaCaT; Cell Culture Facility, Medical University of Graz, Austria), hVFF, human umbilical vein endothelial cells (HUVEC; Lonza, Cat.No. C2519AS) and human monocytic leukemia cell line (THP-1; TIB-202, ATCC, RRID:CVCL_0006) were harvested using the QIAZOL Lysis Reagent. Total mRNA of all cell lysate samples was isolated using the miRNeasy Mini Kit (Qiagen) according to the manufacturer’s instructions. Purified RNA was eluated in RNase-free water and concentration was determined using the NanoDrop 2000c spectrophotometer (Thermo Scientific). Reverse transcription (RT), as well as RT quantitative PCR (RT-qPCR), were performed as previously described [6]. Primer sequences for cell-type specific marker genes are provided in Table 3.

**Table 3:** Primer sequences used for RT-qPCR for cell-type specific characterization.

Gene	Gene symbol	Forward primer	Reverse primer
Beta-2-microglobulin	B2M	AGGCTATCCAGCGTACTCCA	CGGATGGATGAAACCCAGACA
C-C motif chemokine receptor	CCR2	CAAGCCACAAGCTGAACAGAG	CTTCACCGCTCTCGTTGGTA
Cadherin 1	CDH1	TGGTTCAGATCAAATCCAACAAAGA	CTGTCACCTTCAGCCATCCT
Cytokeratin 13	CK13	ATGGTTCCACACCAAGAGTGC	CGTGATCTCTGTCTTGCTGGT
Cytokeratin 14	CK14	ATCCAGAGATGTGACCTCCTC	CTCAGTTCTTGGTGCGAAGG
Cytokeratin 5	CK5	CCAAGGTTGATGCACTGATGG	TGTCAGAGACATGCGTCTGC
Involucrin	IVL	CCTTACTGTGAGTCTGGTTGACA	GGAGGAGGAACAGTCTTGAGG
Thy-1 cell surface antigen	THY-1	TCTCCTGCTAACAGTCTTGCA	CACGGGTCAGGCTGAACTCG
Tumor protein p63	Tp63	CCACCTGGACGTATTCCACTG	TCGAATCAAATGACTAGGAGGGG
Ubiquitously expressed transcript protein	UXT	GCAGCGGGACTTGCGA	TAGCTTCCTGGAGTCGCTCA
Vimentin	VIM	GGACCAGCTAACCAACGACA	TCCTCCTGCAATTTCTCCCG
Von Willebrand factor	VWF	CTCATCGCAGCAAAAGGAGC	ATGCTCATGCACTCCAGGTC

For analyses, C_T_ values of technical triplicates were averaged and relative quantification of all mRNAs of interest was performed based on the 2^-ΔΔC^_T_ method [7] with slight modifications: C_T_ values were normalized to the geometric mean of the internal reference genes beta-2 microglobulin (B2M) and ubiquitously expressed transcript protein (UXT).

#### Histological analysis

Sections of VF and BM tissue from 3 different donors and 3 CC samples were stained with routine H&E staining. Briefly, slides were incubated for 30 minutes at 65°C to soak the paraffin followed by processing with descending alcohol series and by 2 minutes incubation with hematoxylin. After rinsing slides in hot running tap water for 3 minutes, incubation in eosin solution was performed for 2 minutes. Then, slides were shortly dehydrated with ascending alcohol series and coverslipped. Further staining was performed in collaboration with the Diagnostic and Research Institute of Pathology, Medical University of Graz with automated protocols after deparaffinization at 60°C for one hour, followed by processing with DAKO OMNIS (Agilent, Santa Clara, California, USA) for all used antibodies (listed in Table 4).

**Table 4:** Used antibodies for histological staining.

Antibody	Clone	Company	Dilution	Pretreatment	Detection system
CK5/14	-	Biocare, API3026AAk	ready-to-use	Omnis low pH	Omnis Flex Kit HRP; DAB
E-cadherin	NCH-38	Dako, #GA059	ready-to-use	Omnis high pH	Dako Flex Kit; DAB
Involucrin (IVL)	-	Abcam, #ab53112	1:200	CC1 standard	Ventana ultraView; DAB
p63	DAK-p63	Omnis, #GA662	ready-to-use	Omnis low pH	Omnis Flex Kit HRP; DAB
VIM	V9	Dako, #GA630	ready-to-use	Omnis low pH	Omnis Flex Kit HRP; DAB

Immunofluorescence staining for basal collagen type IV (COL IV) was performed on deparaffinized sections after rehydration with descending alcohol series. Antigen retrieval was performed for 15 minutes at 37°C in a water bath using 0.1% Proteinase (Sigma Aldrich), followed by 2 washing steps with phosphate buffered saline (PBS, Thermo Fisher Scientific) for 2 minutes each. Non-specific binding was blocked in a humidified chamber for 45 minutes using a blocking solution (PBS, 0.3% Triton-X.100, Lactan, Graz, Austria), 5% goat serum (Biolabs, San Diego, California, USA). Primary antibody (monoclonal mouse anti-human anti-Collagen Type IV, clone COL-94, Sigma Aldrich, 1:2000) was incubated overnight at 4°C followed by 3 washing steps with PBS for 10 minutes each. Incubation with secondary antibody (goat anti-mouse IgG, Alexa Fluor 594, Cell Signaling, Danvers, Massachusetts, USA, 1:1000) was performed light protected for 1 hour at room temperature. After 3 washing steps with PBS for 10 minutes each, sections were counterstained with Hoechst (Thermo Fisher Scientific, 1:1000) light protected for 15 minutes, further washed with PBS 3 times for 10 minutes each, airtight covered with mounting medium (Dako, Santa Clara, California, USA) and coverslipped.

Analysis of slides were performed on an Olympus IX52 inverse microscope (Olympus, Tokyo, Japan) using the CellSens Standard 1.8.1. imaging software.

#### Proteomic analysis

Snap-frozen samples of 3 CC samples were lysed in 100 μl of 100 mM Tris-HCl (1% sodium dodecyl sulfate (SDS)); 10 mM Tris(2-carbocylethyl)phospine hydrochloride (TCEP); 40 mM chloroacetamide (CAA), (all Sigma Aldrich) by sonication for 2 minutes on ice. CC were subjected to filter aided sample preparation as described previously [6] with minor modifications using 3kD cut off ultracentrifugation filters (Merck Millipore, Burlington, Massachusetts, USA). Buffer was exchanged 2 times with 8 M Urea, 100 mM Tris-HCl (pH 8.5) and once with 2 M Urea, 100 mM Tris-HCl (pH 8.5). After predigestion for 4 hours at 37°C with rLysC (1:100; Promega, Madison, Wisconsin, USA), samples were diluted to 1 M Urea with 100 mM ammonium bicarbonate and digested with trypsin (1:50, Promega) overnight. Peptides were collected by centrifugation at 14,000 x g for 45 minutes at room temperature. Snap-frozen samples of 3 native VF tissue samples were homogenized with steel beads (Sigma Aldrich) using the MagNA lyser instrument for 3 minutes in 600 μl of 100 mM Tris-HCl containing 1% SDS, 10 mM TCEP and 40 mM CAA. After removal of the beads, homogenates were heated up for 10 minutes at 95°C, and centrifuged at 15,000 x g for 7 minutes at 4°C. Protein concentration was determined using the BCA-RAC assay (Thermo Fisher Scientific) according to the manufacturer´s instructions. For Liquid Chromatography—Tandem Mass Spectrometry (LC-MS/MS) analysis, 100 μg of solubilized protein were precipitated with four volumes of acetone overnight and obtained protein pellets were re-solubilized in TFE buffer (25% TFE, 100 mM Tris-HCl, pH 8.5), then diluted with 50 mM ammonium bicarbonate (to reach 10% TFE). Next, diluted samples were predigested with rLysC (Promega, USA, enzyme/protein 1:100) for 4 hours at 37°C and, lastly, digested overnight with trypsin (Promega, enzyme/protein 1:50). For desalting, 3 μg of all digests or half of CC samples were brought to a volume of 100 μl with 1% trifluoroacetic acid in water and loaded onto self-packed 2-layer (18 gauge) 200 μl SDS-RPS Stage Tips (Empore SPE Disks, Sigma Aldrich). The samples were passed through the stage tips by centrifugation for 5 minutes at 1,500 x g at room temperature, washed with 0.2% trifluoroacetic acid, eluted with 5% NH_4_OH/80% acetonitrile into a new tube and dried using a vacuum concentrator. 500 ng protein-digest or 1/100 of CC of each sample were injected resulting in similar total ion chromatrogram intensities. Protein-digests were separated by nano-HPLC (Dionex Ultimate 3000, Thermo Fischer Scientific) equipped with an Aurora nanocolumn (C18, 1.6 μm, 250 x 0.075 mm, Ion Optics, Waltham, Massachusetts, USA) at a flow rate of 300 nl/min at 50°C using the following gradient, where solvent A is 0.1% formic acid (Thermo Fisher Scientific) in water and solvent B is acetonitrile (VWR International, Radnor, Pennsylvania, USA) containing 0.1% formic acid: 0–18 min: 2% B; 18–100 min: 2–25% B; 100–107 min: 25–35% B; 107–108 min: 35–95% B; 108–118 min: 95% B; 118–118 min: 95–2% B; 118–133 min: 2% B. The maXis II ETD mass spectrometer (Bruker Daltonics, Germany) was operated with the captive source in positive mode employing following setting: mass range: 200–2000 m/z, 2 Hz, capillary 1600 V, dry gas flow 3 l/min with 150°C, nanoBooster 0.2 bar, precursor acquisition control top 20 (collision induced dissociation (CID)). The LC-MS/MS data were analysed by MaxQant by searching the public Swissprot human database plus rat collagens (13202466 residues, 20304 sequences) and common contaminants. Carbamidomethylation on Cys was entered as fixed modification, oxidation on methionine as variable modification. Detailed search criteria: trypsin, max. missed cleavage sites: 2; search mode: MS/MS ion search with decoy database search included; precursor mass tolerance: ± 0.006 Da; product mass tolerance: ± 80 ppm; acceptance parameters for identification: 1% peptide-spectra-matches (PSM) false discovery rate (FDR), 1% protein FDR. Additionally, a label free quantification (LFQ) was performed using MaxQuant [8] requiring a minimum of 2 ratio counts of quantified unique peptides. The mass spectrometry proteomics data have been deposited to the ProteomeXchange Consortium via the PRIDE [9] partner repository and can be retrieved via the dataset identifier PXD033688.

#### Statistical analysis

Generation of CC was done in biological triplicates in technical duplicates. Analysis for qPCR data was performed using Excel and Graph Pad Prism 7.0 software (La Jolla, CA, USA). Proteomic data was analyzed by Perseus software version 1.6.6.0. Student’s t-test with Benjamini Hochberg correction (FDR = 0.5%) was used to determine significant differences in the protein repertoire of VF and CC samples for proteomic analyses. A p-value of < 0.05 was determined as statistically significant and results are presented as mean ± standard deviation (SD).

## Results

### Human buccal epithelial cells (hBEC)

#### Isolation procedure

**Fig 2:**
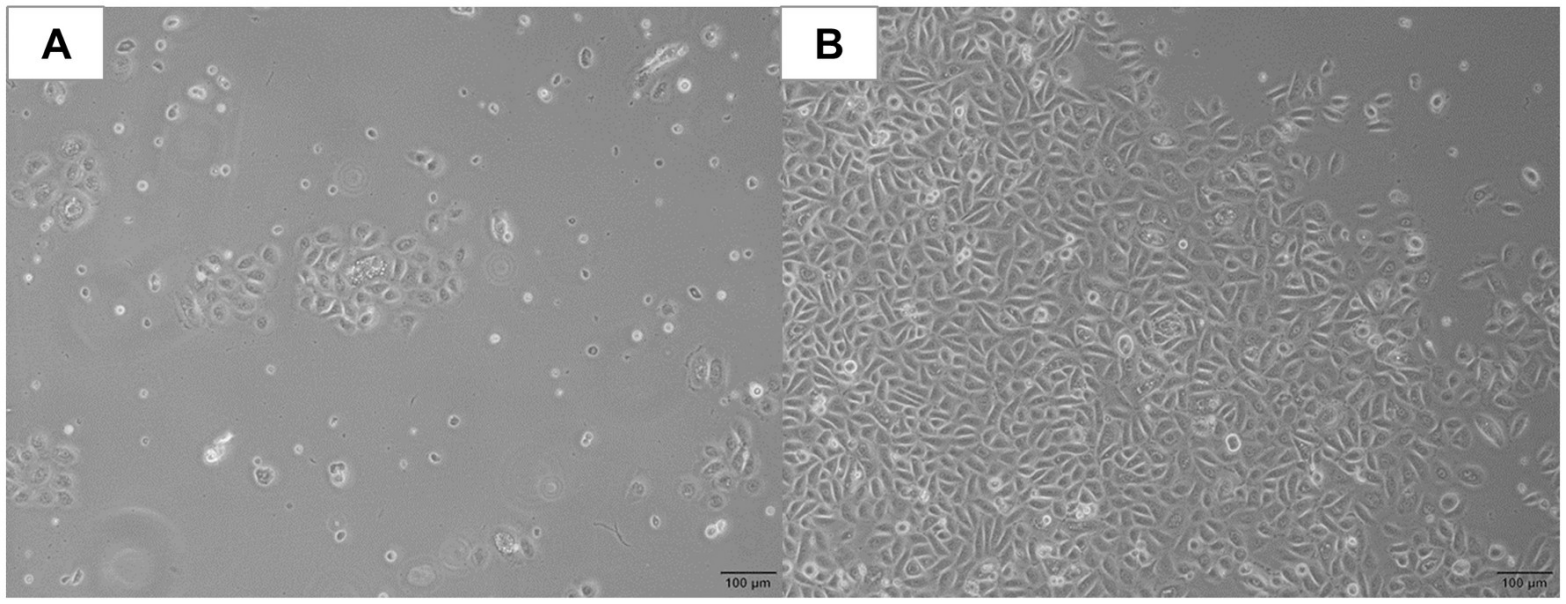
Representative phase-contrast images of isolated hBEC colonies. Cobblestone-like cells 24 hours after start of the isolation (A) and confluent cell layer after additional 14 days of cultivation (B). (both 4x magnification).

One day after starting the isolation procedure, cobblestone-like cells started to form multiple colonies (Fig 2A). Over an additional cultivation period of approximately 14 days, a confluent cell layer was observed (Fig 2B). Cells from four different donors could be passaged and further cultivated.

#### Descriptive cell-type specific characterization

Descriptive characterization of isolated hBEC (n = 4) was performed with RT-qPCR for cell-type specific marker genes, whereas cDNA from HaCaT, hVFF, HUVEC, and THP-1 were used as negative and positive markers.

**Fig 3:**
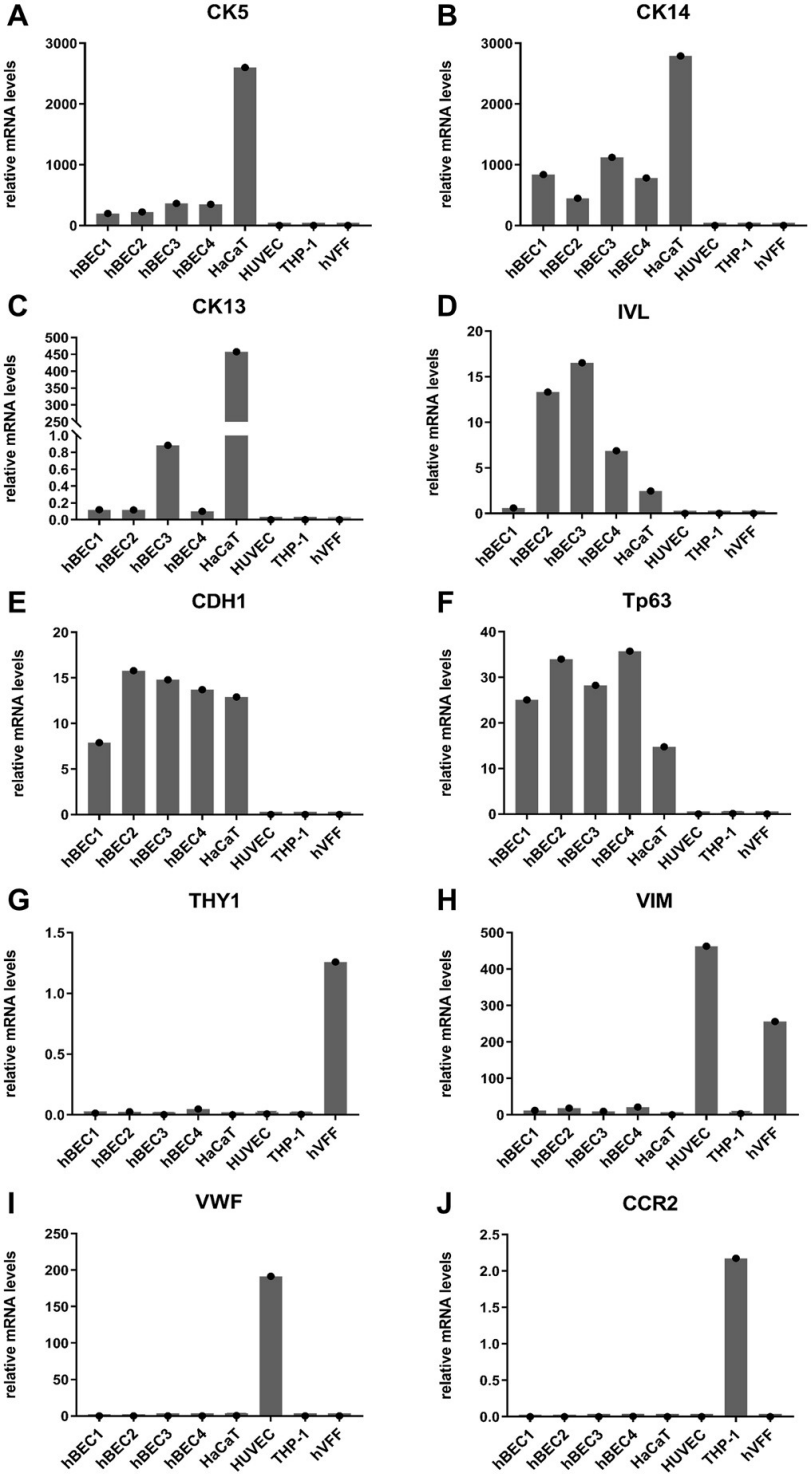
Descriptive hBEC cell characterization. Relative mRNA levels of cell-type specific marker genes cytokeratin 5 (A), cytokeratin 14 (B), cytokeratin 13 (C), involucrin (D), cadherin 1 (E), tumor protein p63 (F), Thy-1 cell surface antigen (G), vimentin (H), von Willebrand factor (I), and C-C motif chemokine receptor (J) in isolated hBEC (n = 4), HaCaT, HUVEC, THP-1, and hVFF cells.

The isolated hBEC and HaCaT cells showed the highest expression of epithelial cell markers cytokeratins (CK) 5 and 14 (Fig 3A and 3B, respectively), whereas HaCaT cells expressed these cytokeratins at an approximately 5 to 10fold higher level than hBEC cells. CK 13 expression was marginally detected in isolated hBEC but approximately 400fold higher in HaCaT cells and was not detected in all other used cells (Fig 3C). Involucrin (IVL) was expressed in both hBEC and HaCaT cells with higher expression in three of the four isolated hBEC cells (Fig 3D). Furthermore, a similar expression level of the epithelial cell marker cadherin 1 (CDH1) was found in all hBEC and HaCaT cells (Fig 3E). Tumor protein p63 (Tp63) was expressed in both hBEC and HaCaT cells with higher expression in all hBEC cells (Fig 3F). Immortalized hVFF showed the highest expression of the fibroblast marker gene Thy-1 cell surface antigen (THY-1), while the expression of the fibroblast/endothelial cell marker vimentin (VIM) was highest in HUVECs followed by hVFF cells (Fig 3G and H, respectively). The endothelial cell marker von Willebrand factor (VWF) was highly expressed in HUVEC cells (Fig 3I), and C-C motif chemokine (CCR2), a monocyte cell marker, was only detected in THP-1 cells (Fig 3J).

### 3D co-cultivation of hBEC and hVFF

**Fig 4:**
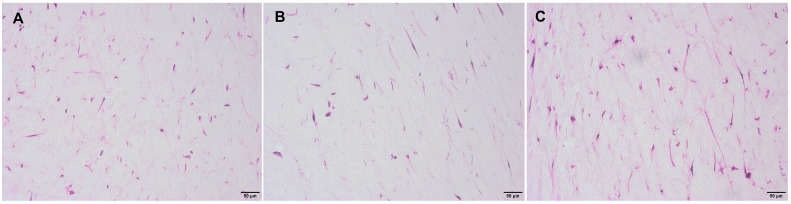
Representative H&E staining images of embedded hVFF. hVFF after a cultivation period of 14 days seeded at a density of 5x10^4^ cells/insert (A), 1x10^5^ cells/insert (B), and 2x10^5^ cells/insert (C). (all 20x magnification).

Histological H&E staining of the rat tail collagen matrix with different hVFF concentrations (5x10^4^, 1x10^5^, and 2x10^5^ cells/insert) showed no obvious difference in cell density (Fig 4A–4C). Cells with the highest seeding concentration (2x10^5^ cells/insert) showed the most intense typical fibroblast-like elongated processes (Fig 4C).

**Fig 5:**
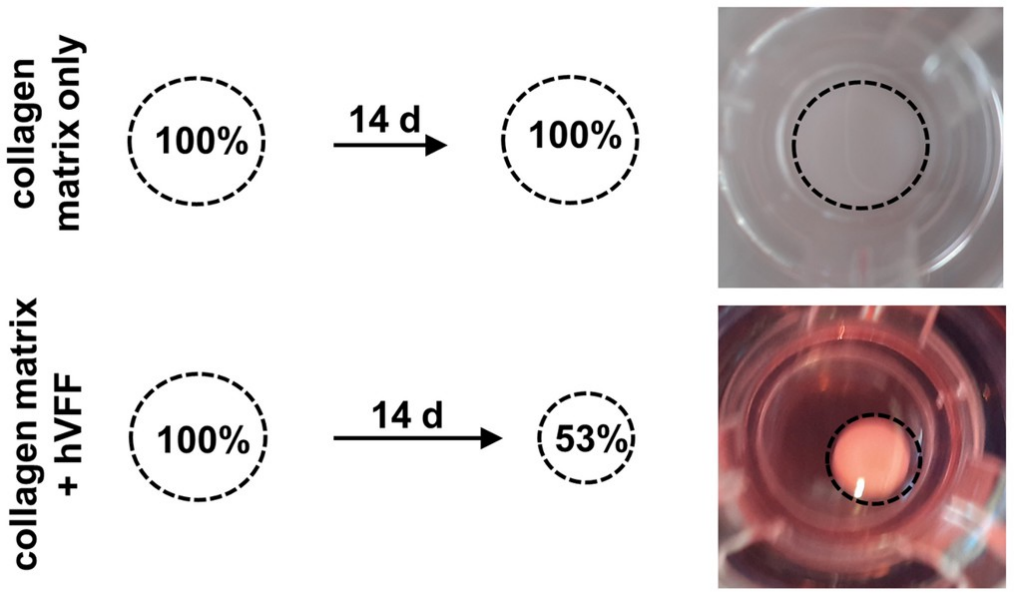
Contraction of rat tail collagen matrix with or without embedded hVFF. Representative schematics and photographs illustrating the matrix contraction following seeding of immortalized hVFF within the rat tail collagen, type I matrix.

A matrix contraction was observed over the first four days of culture and reached a stable averaged plateau of ~53% of initial cross-sectional area compared to the collagen matrix without fibroblasts. No further shrinkage occurred during the following cultivation period for all tested concentrations of seeded hVFF within the collagen matrix (Fig 5).

Combined results from histology and matrix contraction favoured the use of the highest tested hVFF concentration (2x10^5^ cells/insert) for the co-cultivation experiments.

For all co-cultivation experiments, microscopic examination of hVFF within the matrix was only possible during the first steps of the cultivation period. After seeding of hBEC on top of the matrix, cellular components were not entirely visible under the microscope and further growth of cells could not be observed until the harvest of the constructs.

### Bioengineered constructs versus native VF and BM

#### Histological data

**Fig 6:**
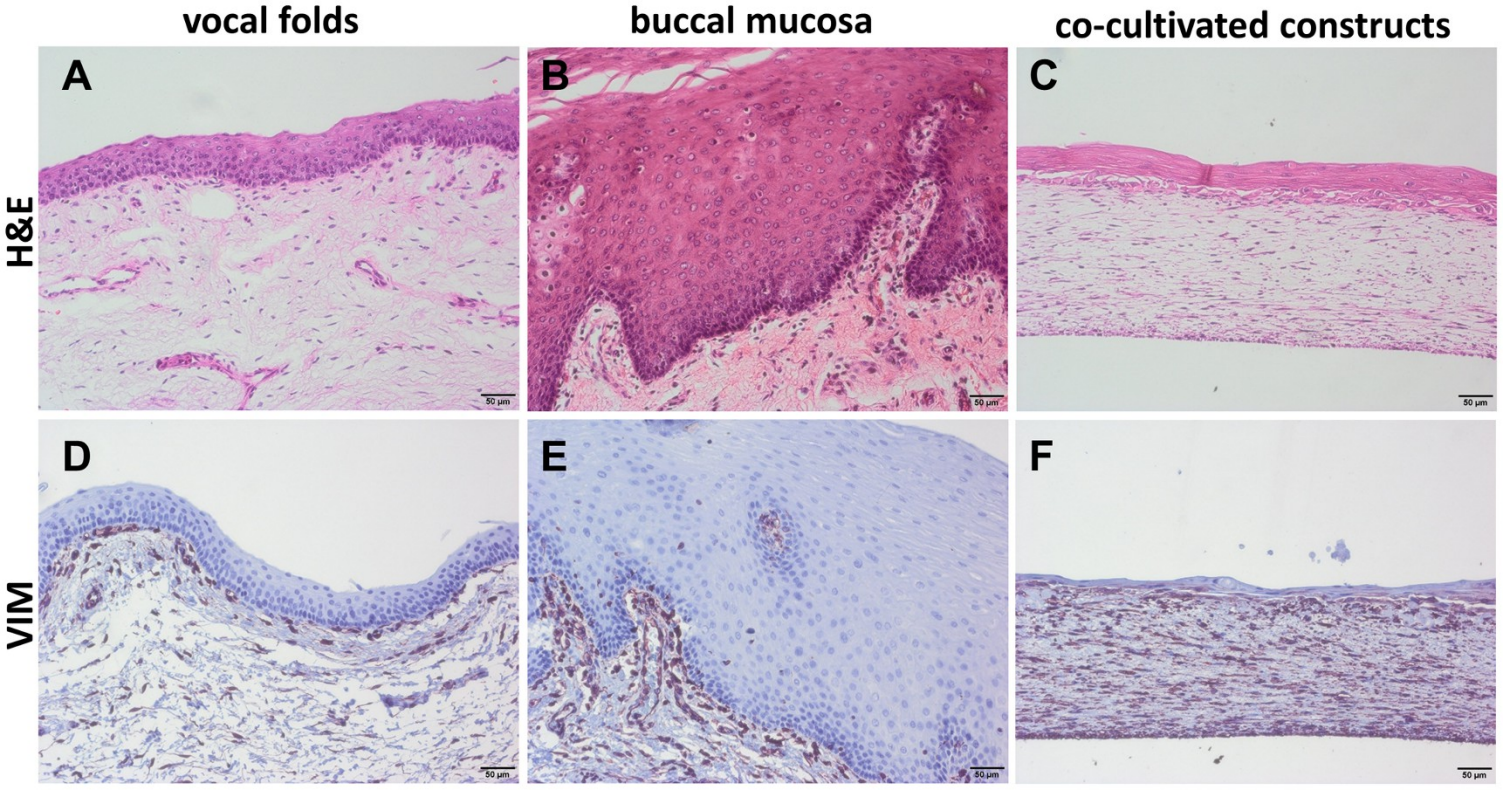
Co-cultivated constructs resemble a multi-layered epithelium and an underlying lamina propria. Representative H&E staining of vocal folds (A), buccal mucosa (B) and co-cultivated construct (C), vimentin staining of vocal folds (D), buccal mucosa (E) and co-cultivated construct (F). All formalin-fixed, paraffin-embedded sections, 20x magnification.

The engineered CC of 3 biological replicates reassembled into a mucosa-like structure, comparable to the native VF after a total cultivation period of 35 days. Key morphologic characteristics including a multi-layered ~20 μm thick stratified epithelium and an underlying, sparsely cell populated lamina propria could be identified. H&E staining (Fig 6A–6C, respectively) and fibroblast-specific staining for vimentin (Fig 6D–6F, respectively) showed an appropriate density and distribution of fibroblasts indicating a successful growth of cells in the engineered CC, similar to native VF and BM samples. Morphologically, most cells showed spindle to star-like somata and elongated processes.

**Fig 7:**
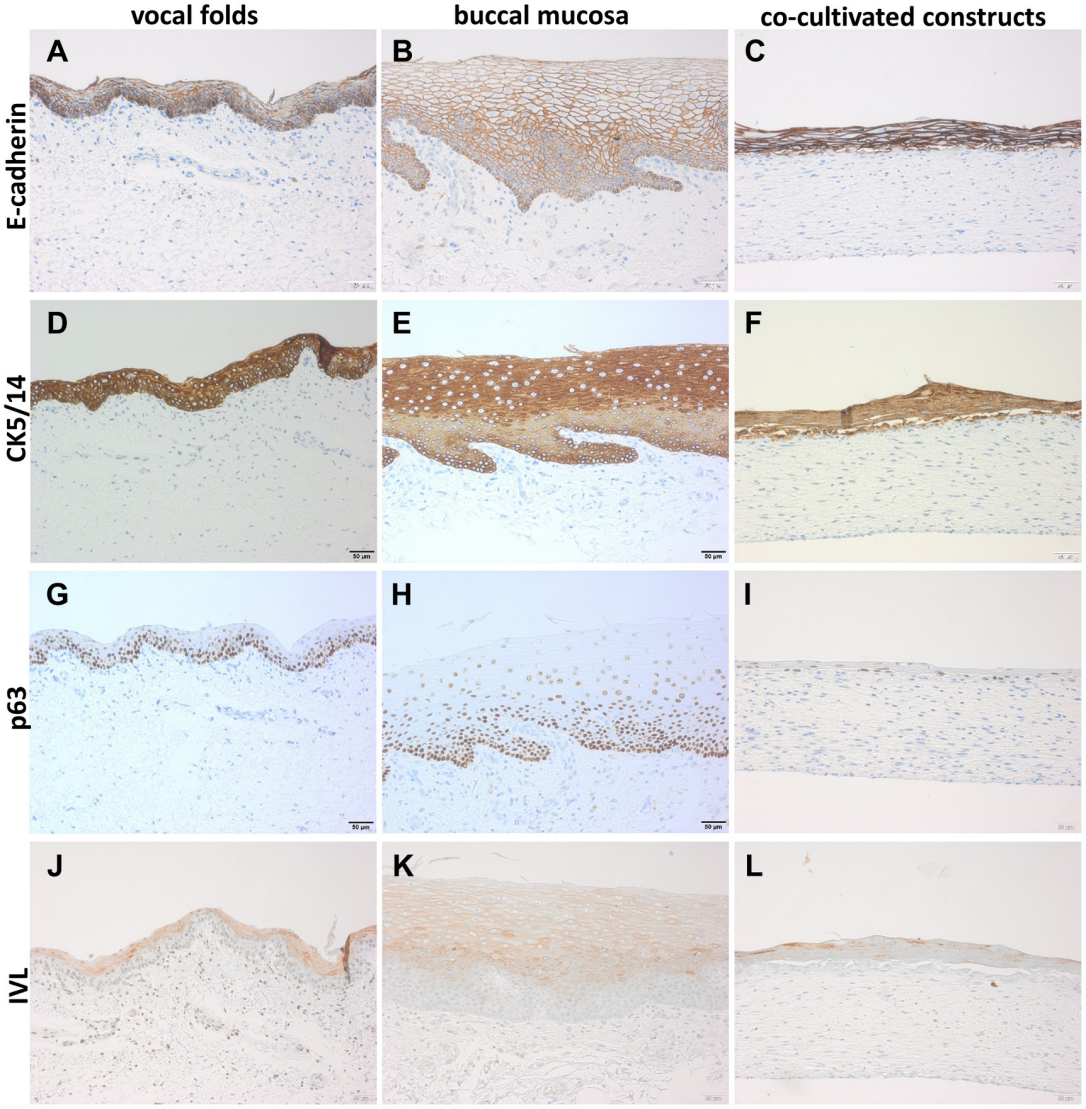
Co-cultivated constructs show adherent cell junctions and multiple epithelial layers. Representative staining for E-cadherin staining of vocal folds (A), buccal mucosa (B) and co-cultivated construct (C); CK5/14 staining of vocal folds (D), buccal mucosa (E) and co-cultivated construct (F); p63 staining of vocal folds (G), buccal mucosa (H) and co-cultivated construct (I); and IVL staining of vocal folds (J), buccal mucosa (K) and co-cultivated construct (L). All formalin-fixed, paraffin-embedded sections, 20x magnification.

Immunohistochemical staining for E-cadherin revealed adherent cell junctions in the native VF and BM samples (Fig 7A and 7B) and also confirmed the re-establishment of junctions in the epithelial layers of the engineered CC (Fig 7C). Morphologically, the construct resembled a structure comparable to the native VF sample, but the epithelial cells appeared more irregular in size. Staining for CK5/14 showed multiple epithelial layers for all three types of samples (Fig 7D–7F). Stratification of epithelial cell layers could be visualized with staining for p63 as basal cell marker in VF, BM and CC samples (Fig 7G–7I, respectively) and with staining for IVL as suprabasal cell marker in VF, BM and CC samples (Fig 7J–7L, respectively).

**Fig 8:**
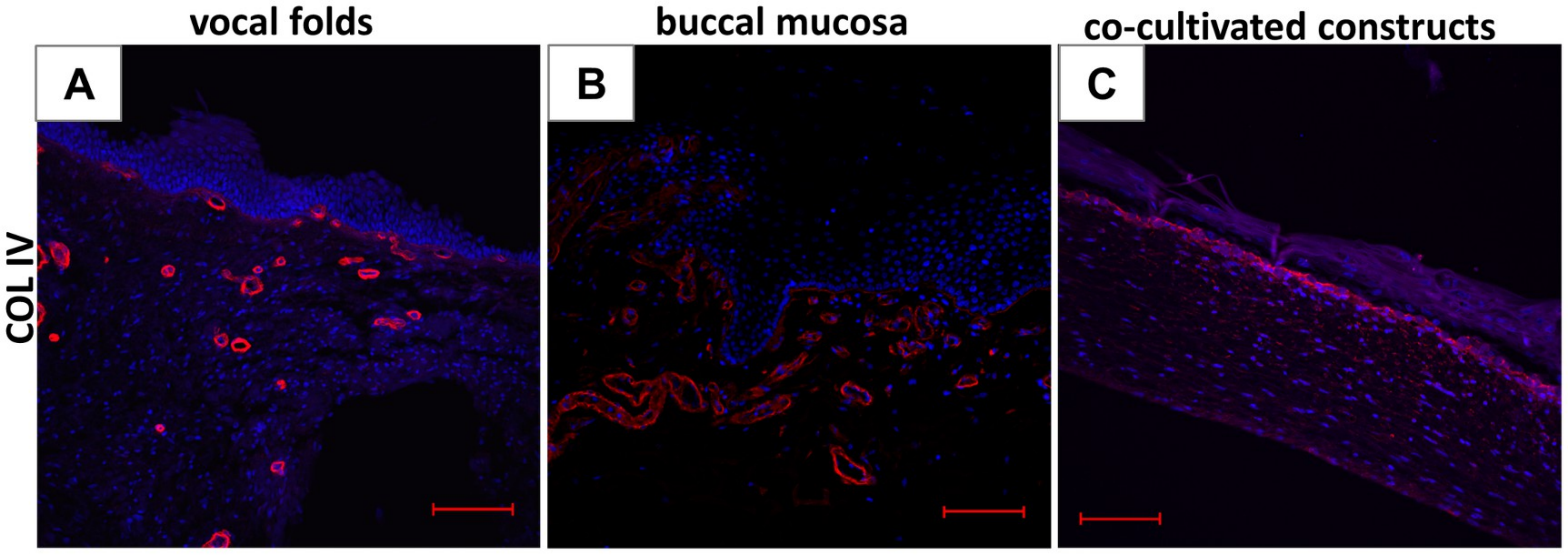
Co-cultivated constructs resemble basement membrane-like structure. Basal collagen type IV (COL IV) staining of formalin-fixed, paraffin-embedded section of vocal folds (A), buccal mucosa (B) and co-cultivated construct (C). (scale bar: 100 μm, all 10x magnification).

Staining for the basement membrane marker, basal COL IV, showed a basement membrane and underlying vascular basement membrane structures within the native VF tissue (Fig 8A) and the native BM tissue (Fig 8B), whereas an assembled barrier-like structure was detected within the engineered CC (Fig 8C).

#### Proteomic data

**Fig 9:**
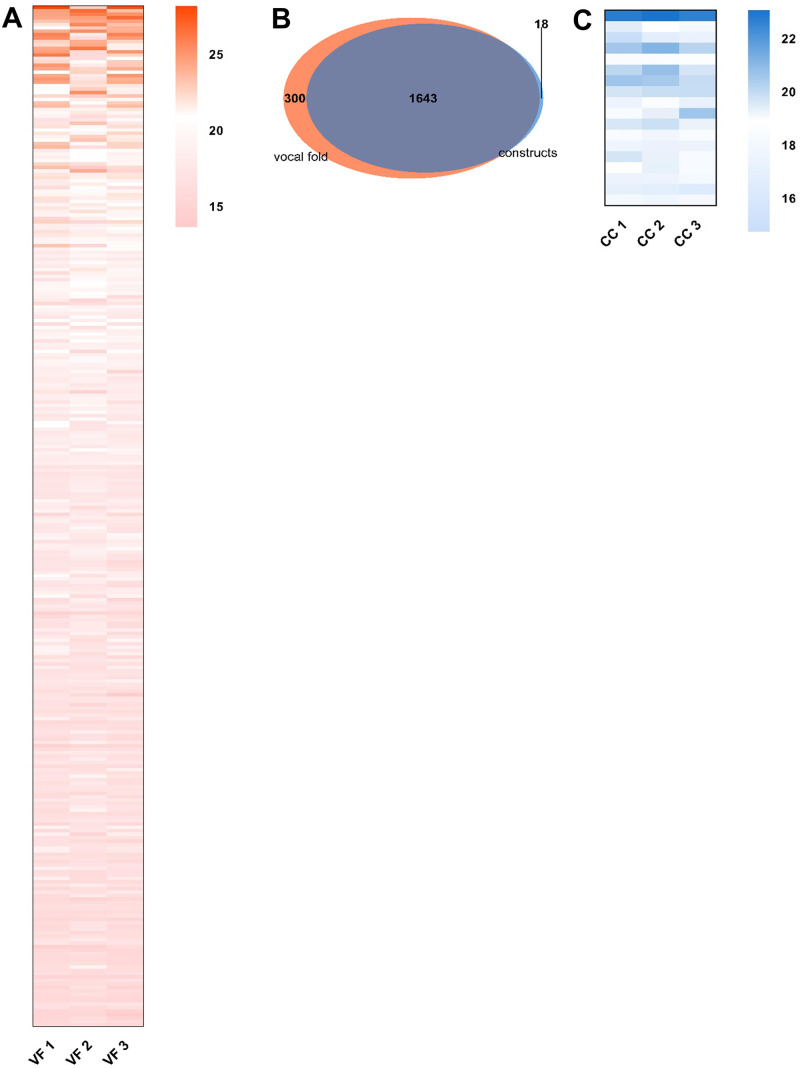
Proteomic profiling of vocal fold and co-cultivated construct samples. Heatmap showing proteins detected solely in VF tissue samples (A). Venn diagram depicting the number of proteins detected solely in VF tissue samples, solely in CC samples, and in both sample types (B). Heatmap showing proteins detected solely in CC samples (C). Proteins are sorted in a decreasing manner according to their mean LFQ intensity values (A,C). Protein IDs, protein and gene names are listed in S4 and S5 Tables. Data from 3 biological replicates of VF and CC samples.

We identified 3171 proteins with 130,782 high-resolution CID-MS/MS protein matched spectra in total after the database search. After removing proteins identified only by site, reverse hits, and potential contaminants, 20,619 unique peptides corresponding to 3072 proteins (S2 Table) were identified with a FDR <1% using Perseus version 1.6.6.0. After filtering for peptide counts (razor + unique peptides) >1, and 3 valid values in at least one sample group (VF or CC), we identified a total of 1961 proteins (1624 proteins on average, SD = 287.7). Of these, 1643 (83.8%, Fig 9B and S3 Table) were detected in CC and VF, 300 proteins (15.3%, Fig 9A and S4 Table) were identified only in VF and classified with GO enrichment analysis using the STRING (Search Tool for Retrieval of Interacting Genes/Proteins) database [10] (S5 Table), and 18 proteins (0.9%, Fig 9C and S6 Table) were found only in CC samples and classified with GO enrichment analysis (S7 Table).

**Fig 10:**
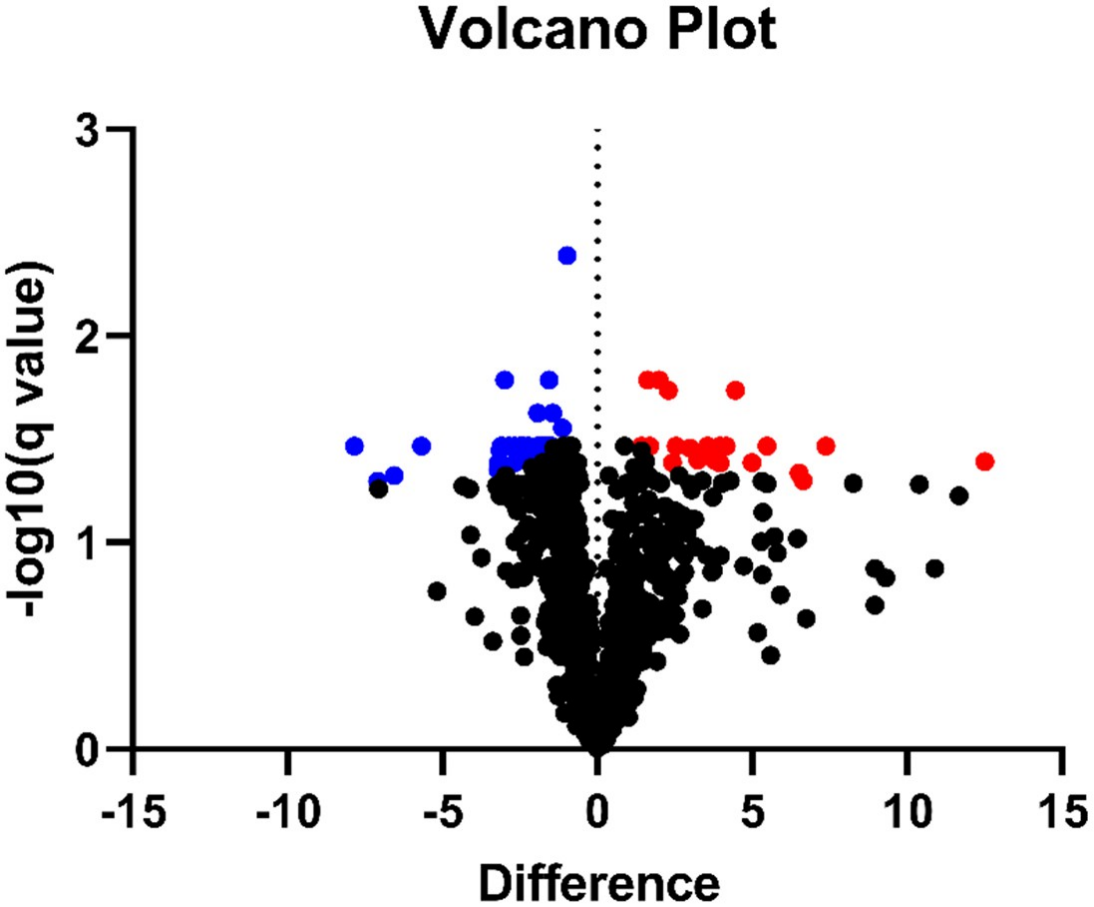
Differentially expressed proteins in vocal fold and co-cultivated construct samples. Volcano plot showing 893 identified proteins including 53 proteins with significantly differential protein abundance (data from 3 biological replicates of VF and CC samples, p = 0.005) after Benjamini-Hochberg correction for multiple testing between co-cultivated construct (CC, left side, blue dots) and vocal fold (VF, right side, red dots) samples.

To further compare the detected protein repertoire of 1643 proteins in both sample types, log2 transformed LFQ intensity values were filtered for 3 valid values in each group (VF and CC). The 893 identified proteins (S8 Table) were then subjected to a paired t-test (original FDR method of Benjamini and Hochberg, FDR = 0.5%). The analysis showed a statistically significant differential expression of 53 proteins. Of those, 21 (5.9%) were upregulated in VF and 32 (3.6%) were upregulated in CC samples (Fig 10 and S9 Table).

#### Proteins with different abundance

To identify the most distinct biological categories between the two sample types, classification of proteins that were significantly upregulated in VF or CC samples was performed by GO enrichment analysis using the STRING database. In total, 7 gene sets were found to be significantly enriched in VF: “protein-containing complex binding”, “structural molecule activity” and “extracellular matrix structural constituent” (GO molecular function), as well as “extracellular region”, “extracellular space”, “extracellular exosome” and “collagen-containing extracellular matrix” (GO cellular component) (Table 5).

**Table 5:** Gene sets significantly enriched in vocal fold versus co-cultivated construct samples.

Term ID	Term name	Count	strength	FDR	Matching proteins
**Molecular Function**					
GO:0044877	Protein-containing complex binding	8	0.81	0.0235	MYH2,COL14A1,NUMA1,LAMB2,ANXA11,TNXB,TAGLN,EPPK1
GO:0005198	Structural molecule activity	6	0.97	0.0339	COL14A1,PRELP,NUMA1, LAMB2,TNXB,EPPK1
GO:0005201	Extracellular matrix structural constituent	4	1.52	0.0215	COL14A1,PRELP,LAMB2,TNXB
**Cellular Component**					
GO:005576	Extracellular region	14	0.52	0.0025	ALDOC,ALDH2,F13A1,ALDH1A1,COL14A1,PRELP,GOT1, DNAJC9,NUMA1,LAMB2, ANXA11,TNXB,GSTK1,FAM213A
GO:0005615	Extracellular space	13	0.6	0.0025	ALDOC,ALDH2,F13A1,ALDH1A1,COL14A1,PRELP,GOT1, DNAJC9,NUMA1,LAMB2, ANXA11,TNXB,GSTK1
GO:0070062	Extracellular exosome	10	0.6	0.0047	ALDOC,ALDH2,ALDH1A1, PRELP,GOT1,NUMA1,LAMB2, ANXA11,TNXB,GSTK1
GO:0062023	Collagen-containing extracellular matrix	6	1.17	0.0025	F13A1,COL14A1,PRELP,LAMB2,ANXA11,TNXB

For CC, 15 enriched gene sets were identified: “cell adhesion molecule binding” and “cadherin binding” (GO molecular function), as well as “cytoplasm”, “extracellular region”, “extracellular space”, “vesicle”, “extracellular exosome”, “cytosol”, “cytoplasmic vesicle”, “collagen-containing extracellular matrix”, “melanosome”, “basement membrane”, “proteasome complex”, “laminin complex”, and “proteasome core complex” (GO cellular component) (Table 6).

**Table 6:** Gene sets significantly enriched in co-cultivated construct versus vocal fold samples.

Term ID	Term name	Count	strength	FDR	Matching proteins
**Molecular Function**					
GO:0050839	Cell adhesion molecule binding	8	0.96	0.0065	PLIN3,PRDX1,S100P,LAMA3, SFN,DSP,ITGB1,RAN
GO:0045296	Cadherin binding	6	1.04	0.0261	PLIN3,PRDX1,S100P,SFN, ITGB1,RAN
**Cellular Component**					
GO:0005737	Cytoplasm	32	0.23	8.33e-06	PLIN3,DSTN,TXNDC17, SERPINF1,PSMA6,PRDX1, LGALS3BP,LAMC2,PSMD3,PML,FARSB,PSMB4,S100P,ANPEP, GLUL,LAMA3,SFN,HNRNPAB, MAP4,EIF3B,IFIT1,YARS,ACOT7,SRPX,LGALS7,DSP,SERPINB1, TYMP,ITGB1,VSNL1,PDCD6IP, RAN
GO:0005576	Extracellular region	24	0.55	4.55e-08	DSTN,TXNDC17,SERPINF1, PSMA6,PRDX1,LGALS3BP, LAMC2,PSMD3,PSMB4,S100P, ANPEP,GLUL,LAMA3,SFN, EIF3B,YARS,ACOT7,SRPX, LGALS7,DSP,SERPINB1,ITGB1,PDCD6IP,RAN
GO:0005615	Extracellular space	23	0.64	2.17e-09	DSTN,TXNDC17,SERPINF1, PSMA6,PRDX1,LGALS3BP, LAMC2,PSMD3,PSMB4,S100P, ANPEP,GLUL,LAMA3,SFN, EIF3B,YARS,ACOT7,LGALS7, DSP,SERPINB1,ITGB1, PDCD6IP,RAN
GO:0031982	Vesicle	23	0.56	8.60e-08	PLIN3,DSTN,TXNDC17, SERPINF1,PSMA6,PRDX1, LGALS3BP,PSMD3,PML,PSMB4,S100P,ANPEP,GLUL,LAMA3, SFN,EIF3B,ACOT7,LGALS7, DSP,SERPINB1,ITGB1, PDCD6IP,RAN
GO:0070062	Extracellular exosome	21	0.79	3.19e-10	DSTN,TXNDC17,SERPINF1, PSMA6,PRDX1,LGALS3BP, PSMD3,PSMB4,S100P,ANPEP, GLUL,LAMA3,SFN,EIF3B, ACOT7,LGALS7,DSP,SERPINB1,ITGB1,PDCD6IP,RAN
GO:0005829	Cytosol	19	0.35	0.0114	PLIN3,TXNDC17,PSMA6,PRDX1,PSMD3,PML,FARSB,PSMB4, GLUL,SFN,MAP4,EIF3B,IFIT1, YARS,ACOT7,TYMP,VSNL1, PDCD6IP,RAN
GO:0031410	Cytoplasmic vesicle	13	0.52	0.0070	PLIN3,SERPINF1,PRDX1, LGALS3BP,PSMD3,PML,S100P,ANPEP,DSP,SERPINB1,ITGB1, PDCD6IP,RAN
GO:0062023	Collagen-containing extracellular matrix	6	0.97	0.0070	SERPINF1,LGALS3BP,LAMC2, LAMA3,SRPX,SERPINB1
GO:0042470	Melanosome	5	1.47	0.00018	SERPINF1,PRDX1,ITGB1, PDCD6IP,RAN
GO:0005604	Basement membrane	3	1.28	0.0474	SERPINF1,LAMC2,LAMA3
GO:0000502	Proteasome complex	3	1.47	0.0183	PSMA6,PSMD3,PSMB4
GO:0043256	Laminin complex	2	2.05	0.0207	LAMC2,LAMA3
GO:0005839	Proteasome core complex	2	1.79	0.0474	PSMA6,PSMB4

## Discussion

Most laryngeal basic research so far has focused on single cell types (primarily VFF), while only little is known about human VF epithelial (hVFE) cells or functional aspects such as the important epithelial-fibroblast crosstalk. As a consequence, many fundamental questions about this important structure remain unanswered. The establishment of an organotypic laryngeal mucosa model could overcome some of these limitations. For example, it could facilitate studies of VF mucosal homeostasis, which is known to be maintained by complex interactions between different cell types, extracellular matrix (ECM) components, and external factors such as vibration [11–15]. Since the complete differentiation of the highly complex human VF mucosa takes more than ten years [16], it has not been fully recapitulated *in vitro* to date, despite important recent developments [2]. While bioengineered mucosa consisting of primary hVFF and hVFE cells provides an optimal setting for an *in vitro* model of the VF mucosa, primary VF mucosal cells are not available for large-scale therapeutics [2]. To overcome the difficulty in procuring VFE cells, Lungova et.al used hiPSCs, transfected via TALEN constructs for green fluorescent protein, which mimics the development of VFE cells in utero [17]. However, the process of hiPSC differentiation is complex and probably not feasible for many research groups.

Since BM epithelial cells are more easily available compared to hVFE, we decided to include this cell type in a 3D co-culture setting with hVFF. This approach was supported by the results of a previous study from our group, which showed a high similarity between mucosal VF and BM tissue at the proteomic level [4]. The VF epithelium is the first cellular layer and consists of five to ten closely packed non-keratinized stratified squamous cell layers, in contrast to keratinized cell layers, such as those found in the skin [18].

Epithelial cells from four different human donors were passaged and further cultivated without any kind of coating of cell culture dishes, in contrast to previously published procedures [3,19–21]. At higher passages (four to six), the cells showed a shift to a spindle-shaped, fibroblast-like morphology. As described for various cell types [19,22,23], this may indicate a possible donor-related replicative senescence that can alter gene expression and metabolism of isolated cells. Few studies have reported the maintenance of primary cell culture characteristics for more than ten generations [24,25]. Therefore, only cells up to passage number three were used for RNA-isolation and CC generation, which was the limiting factor for construct production.

A rat tail collagen-fibroblast matrix was the main structural component of our engineered construct, so we first tested the number, density, and distribution of embedded hVFF. We observed a high and stable matrix contraction (~53% of initial cross-sectional area compared to the collagen matrix without fibroblasts) over the first four days of culture. These results are similar to those of Jettè et al. [26], who used primary hVFF cells as compared to immortalized hVFF in our study.

Atop of the bioengineered constructs, we identified a key morphologic feature of native VF mucosa, namely the presence of multiple epithelial layers with re-established adherens junctions. Morphologically, the epithelial cells of the construct appeared to be more irregular in size. The requirement of both fibroblasts and epithelial cells for the growth of a multilayered epithelium *in vitro* is well-documented in the literature [27,28]. Furthermore, the presence and interplay of both cell types promoted the growth of a continuous basement membrane-like structure between the collagen-fibroblast matrix and the epithelium. In line with results from Pöschl et al. [29], histological staining appeared weaker in the constructs than in the native VF mucosa, indicating an early stage of development and maturation of this specialized extracellular tissue component. In addition, stratification of epithelial cell layers could be visualized by staining for the basal cell marker p63 and the suprabasal cell marker IVL in the bioengineered constructs. Based on the limited histological staining of our constructs, it would be beneficial to prolong the cultivation time of the construct to obtain a more developed mucosal structure in terms of epithelial multilayered and stratified structure and to provide additional staining for functional characterization, e.g. mucus expression.

In addition to the structural similarity, proteomic data confirmed considerable similarities between the constructs and native VF mucosa. The majority of the detected proteins were present in both sample types, although a moderate number of 300 proteins were detected only in the native VF mucosa samples. This is most likely due to the increased dynamic range of the constructs due to the addition of rat collagen and/or the multiple cell types naturally present in the VF mucosa, such as macrophages and endothelial cells in the ECM layer, as well as muscle cells of the underlying vocalis muscle collected during VF mucosa harvesting [30–32]. Accordingly, these proteins are mainly involved in processes such as cellular component biogenesis and organization, and muscle-related biological processes such as muscle contraction, filament sliding or actin-filament-based process.

A more stringent analysis of the proteins found in both sample types identified only 53 proteins with significantly different abundance. Interestingly, several isoforms of laminin, which have been described to be sufficiently stable for the recruitment and overall organization of basement membrane protein aggregates during early development [29,33], were significantly enriched in the constructs. Other major basement membrane components, namely COL IV, isoforms of the glycoprotein nidogen and the heparin sulfate proteoglycan agrin [34], were identified in our constructs and their abundance was similar to that in VF tissue.

In conclusion, our alternative laryngeal mucosa model, based on easily available cell sources, shares many histologic and proteomic characteristics with native human VF mucosa. We are aware that a combination of immortalized hVFF and immortalized hVFE cells would be the best setting for an *in vitro* model for scientific exploration and preclinical laryngeal research or pharmaceutical applications. However, immortalized hVFE cell lines were not available when the experiments were performed [35]. Nevertheless, our model may provide an alternative *in vitro* platform to explore VF biology, including physiology and pathophysiology, since animal experiments should be attempted to be kept at a minimum.

Due to the use of immortalized hVFF and rat tail collagen type I in our constructs, this specific type of construct is not suitable for human tissue replacement. Nevertheless, the use of primary VFF and BEC, in combination with an animal-free matrix may pave the way for a similar type of construct as a graft for VF mucosal replacement/transplantation. The next step will be to extend the cultivation period of the construct to obtain additional histologic analysis and to apply vibration [36] at later points in the cultivation period to better approximate the physiological environment of the VF.

## Supporting information

S1 Table Raw data from descriptive cell characterization. (XLSX)

S2 Table Total proteome list of identified proteins in vocal fold (VF) tissue and co-cultivated construct (CC) samples. 3072 unique proteins were identified after removing proteins identified only by site, reverse hits, and potential contaminants, FDR <1%.
(XLSX)

S3 Table Total proteome list of identified proteins in VF and CC samples. 1643 unique proteins were identified, log2 transformed data.
(XLSX)

S4 Table Total proteome list of identified proteins in VF samples. 300 unique proteins were identified in VF samples, log2 transformed data.
(XLSX)

S5 Table Results of gene ontology enrichment analysis of identified proteins only in VF samples. 300 unique proteins were identified in VF samples.
(XLSX)

S6 Table Total proteome list of identified proteins in CC samples. 18 unique proteins were identified in CC samples, log2 transformed data.
(XLSX)

S7 Table Results of gene ontology enrichment analysis of identified proteins only in CC samples. 18 unique proteins were identified in CC samples.
(XLSX)

S8 Table Total proteome list of identified proteins in VF and CC samples. 893 unique proteins were identified in VF and CC samples after filtering for 3 valid values in each group, log2 transformed data.
(XLSX)

S9 Table Results of paired t-test of identified proteins in VF and CC samples. 53 unique proteins in VF and CC samples were identified through paired t-test (original FDR method of Benjamini and Hochberg, FDR 0.5%, log2 transformed data.
(XLSX)

## References

[1] Fukahori M, Chitose S, Sato K, Sueyoshi S, Kurita T, Umeno H, et al. Regeneration of Vocal Fold Mucosa Using Tissue-Engineered Structures with Oral Mucosal Cells. PLoS One. 2016;11(1):e0146151. doi: 10.1371/journal.pone.0146151 26730600 PMC4701435

[2] Ling C, Li Q, Brown ME, Kishimoto Y, Toya Y, Devine EE, et al. Bioengineered vocal fold mucosa for voice restoration. Sci Transl Med. 2015;7(314):314ra187. doi: 10.1126/scitranslmed.aab4014 26582902 PMC4669060

[3] Chen X, Lungova V, Zhang H, Mohanty C, Kendziorski C, Thibeault SL. Novel immortalized human vocal fold epithelial cell line: In vitro tool for mucosal biology. FASEB J. 2021;35(2):e21243. doi: 10.1096/fj.202001423R 33428261 PMC7839467

[4] Grossmann T, Darnhofer B, Birner-Gruenberger R, Kirsch A, Gugatschka M. Descriptive proteomics of paired human vocal fold and buccal mucosa tissue. Proteomics Clin Appl. 2022;16(2):e2100050. doi: 10.1002/prca.202100050 34792860 PMC9286793

[5] Chen X, Thibeault SL. Novel isolation and biochemical characterization of immortalized fibroblasts for tissue engineering vocal fold lamina propria. Tissue Eng Part C Methods. 2009;15(2):201–12. doi: 10.1089/ten.tec.2008.0390 19108681 PMC2819707

[6] Karbiener M, Darnhofer B, Frisch MT, Rinner B, Birner-Gruenberger R, Gugatschka M. Comparative proteomics of paired vocal fold and oral mucosa fibroblasts. J Proteomics. 2017;155:11–21. doi: 10.1016/j.jprot.2017.01.010 28099887 PMC5389448

[7] Rao X, Huang X, Zhou Z, Lin X. An improvement of the 2^(-delta delta CT) method for quantitative real-time polymerase chain reaction data analysis. Biostat Bioinforma Biomath. 2013;3(3):71–85. 25558171 PMC4280562

[8] Cox J, Hein MY, Luber CA, Paron I, Nagaraj N, Mann M. Accurate proteome-wide label-free quantification by delayed normalization and maximal peptide ratio extraction, termed MaxLFQ. Mol Cell Proteomics. 2014;13(9):2513–26. doi: 10.1074/mcp.M113.031591 24942700 PMC4159666

[9] Deutsch EW, Bandeira N, Sharma V, Perez-Riverol Y, Carver JJ, Kundu DJ, et al. The ProteomeXchange consortium in 2020: enabling ’big data’ approaches in proteomics. Nucleic Acids Res. 2020;48(D1):D1145–D52. doi: 10.1093/nar/gkz984 31686107 PMC7145525

[10] Szklarczyk D, Gable AL, Lyon D, Junge A, Wyder S, Huerta-Cepas J, et al. STRING v11: protein-protein association networks with increased coverage, supporting functional discovery in genome-wide experimental datasets. Nucleic Acids Res. 2019;47(D1):D607–D13. doi: 10.1093/nar/gky1131 30476243 PMC6323986

[11] Leydon C, Imaizumi M, Bartlett RS, Wang SF, Thibeault SL. Epithelial cells are active participants in vocal fold wound healing: an in vivo animal model of injury. PLoS One. 2014;9(12):e115389. doi: 10.1371/journal.pone.0115389 25514022 PMC4267843

[12] Gray SD. Cellular physiology of the vocal folds. Otolaryngol Clin North Am. 2000;33(4):679–98. doi: 10.1016/s0030-6665(05)70237-1 10918654

[13] Hirano M, Sato K, Nakashima T. Fibroblasts in human vocal fold mucosa. Acta Otolaryngol. 1999;119(2):271–6. doi: 10.1080/00016489950181800 10320090

[14] Palencia L, Das A, Palecek SP, Thibeault SL, Leydon C. Epidermal growth factor mediated healing in stem cell-derived vocal fold mucosa. J Surg Res. 2015;197(1):32–8. doi: 10.1016/j.jss.2015.02.066 25818979 PMC4457682

[15] Hiwatashi N, Bing R, Kraja I, Branski RC. Mesenchymal stem cells have antifibrotic effects on transforming growth factor-beta1-stimulated vocal fold fibroblasts. Laryngoscope. 2017;127(1):E35–E41. 27345475 10.1002/lary.26121 PMC5177483

[16] Hartnick CJ, Rehbar R, Prasad V. Development and maturation of the pediatric human vocal fold lamina propria. Laryngoscope. 2005;115(1):4–15. doi: 10.1097/01.mlg.0000150685.54893.e9 15630357

[17] Lungova V, Chen X, Wang Z, Kendziorski C, Thibeault SL. Human induced pluripotent stem cell-derived vocal fold mucosa mimics development and responses to smoke exposure. Nat Commun. 2019;10(1):4161. doi: 10.1038/s41467-019-12069-w 31551422 PMC6760204

[18] Arens C, Glanz H, Wonckhaus J, Hersemeyer K, Kraft M. Histologic assessment of epithelial thickness in early laryngeal cancer or precursor lesions and its impact on endoscopic imaging. Eur Arch Otorhinolaryngol. 2007;264(6):645–9. doi: 10.1007/s00405-007-0246-8 17294207

[19] Morino T, Takagi R, Yamamoto K, Kojima H, Yamato M. Explant culture of oral mucosal epithelial cells for fabricating transplantable epithelial cell sheet. Regen Ther. 2019;10:36–45. doi: 10.1016/j.reth.2018.10.006 30581895 PMC6298907

[20] Erickson-DiRenzo E, Leydon C, Thibeault SL. Methodology for the establishment of primary porcine vocal fold epithelial cell cultures. Laryngoscope. 2019;129(10):E355–E64. doi: 10.1002/lary.27909 30848488 PMC6779414

[21] Johnston N, Yan JC, Hoekzema CR, Samuels TL, Stoner GD, Blumin JH, et al. Pepsin promotes proliferation of laryngeal and pharyngeal epithelial cells. Laryngoscope. 2012;122(6):1317–25. doi: 10.1002/lary.23307 22570308 PMC3816638

[22] Chen X, Thibeault SL. Characteristics of age-related changes in cultured human vocal fold fibroblasts. Laryngoscope. 2008;118(9):1700–4. doi: 10.1097/MLG.0b013e31817aec6c 18677285 PMC2652878

[23] Southgate J, Williams HK, Trejdosiewicz LK, Hodges GM. Primary culture of human oral epithelial cells. Growth requirements and expression of differentiated characteristics. Lab Invest. 1987;56(2):211–23. 2433501

[24] Kaeffer B. Mammalian intestinal epithelial cells in primary culture: a mini-review. In Vitro Cell Dev Biol Anim. 2002;38(3):123–34. doi: 10.1290/1071-2690(2002)038&lt;0123:MIECIP&gt;2.0.CO;2 12026159

[25] Loret S, Rusu D, El Moualij B, Taminiau B, Heinen E, Dandrifosse G, et al. Preliminary characterization of jejunocyte and colonocyte cell lines isolated by enzymatic digestion from adult and young cattle. Res Vet Sci. 2009;87(1):123–32. doi: 10.1016/j.rvsc.2008.12.002 19162286

[26] Jette ME, Hayer SD, Thibeault SL. Characterization of human vocal fold fibroblasts derived from chronic scar. Laryngoscope. 2013;123(3):738–45. doi: 10.1002/lary.23681 23444190 PMC3584344

[27] Leydon C, Selekman JA, Palecek S, Thibeault SL. Human embryonic stem cell-derived epithelial cells in a novel in vitro model of vocal mucosa. Tissue Eng Part A. 2013;19(19–20):2233–41. doi: 10.1089/ten.TEA.2012.0744 23672433 PMC3761441

[28] Yamaguchi T, Shin T, Sugihara H. Reconstruction of the laryngeal mucosa. A three-dimensional collagen gel matrix culture. Arch Otolaryngol Head Neck Surg. 1996;122(6):649–54. doi: 10.1001/archotol.1996.01890180057014 8639298

[29] Poschl E, Schlotzer-Schrehardt U, Brachvogel B, Saito K, Ninomiya Y, Mayer U. Collagen IV is essential for basement membrane stability but dispensable for initiation of its assembly during early development. Development. 2004;131(7):1619–28. doi: 10.1242/dev.01037 14998921

[30] Catten M, Gray SD, Hammond TH, Zhou R, Hammond E. Analysis of cellular location and concentration in vocal fold lamina propria. Otolaryngol Head Neck Surg. 1998;118(5):663–7. doi: 10.1177/019459989811800516 9591866

[31] Boseley ME, Hartnick CJ. Development of the human true vocal fold: depth of cell layers and quantifying cell types within the lamina propria. Ann Otol Rhinol Laryngol. 2006;115(10):784–8. doi: 10.1177/000348940611501012 17076102

[32] Jiang J, Lin E, Hanson DG. Vocal fold physiology. Otolaryngol Clin North Am. 2000;33(4):699–718. doi: 10.1016/s0030-6665(05)70238-3 10918655

[33] Hohenester E, Yurchenco PD. Laminins in basement membrane assembly. Cell Adh Migr. 2013;7(1):56–63. doi: 10.4161/cam.21831 23076216 PMC3544787

[34] Khalilgharibi N, Mao Y. To form and function: on the role of basement membrane mechanics in tissue development, homeostasis and disease. Open Biol. 2021;11(2):200360. doi: 10.1098/rsob.200360 33593159 PMC8061686

[35] Chen X., Lungova V., Zhang H., Mohanty C., Kendziorski C., & Thibeault S. L. (2021). Novel immortalized human vocal fold epithelial cell line: In vitro tool for mucosal biology. FASEB journal: official publication of the Federation of American Societies for Experimental Biology, 35(2), e21243.). doi: 10.1096/fj.202001423R 33428261 PMC7839467

[36] Kirsch A, Hortobagyi D, Stachl T, Karbiener M, Grossmann T, Gerstenberger C, et al. Development and validation of a novel phonomimetic bioreactor. PLoS One. 2019;14(3):e0213788. doi: 10.1371/journal.pone.0213788 30870529 PMC6417646

